# T243. RESOURCE GROUP-ACT: RELATIVES’ PERSPECTIVES

**DOI:** 10.1093/schbul/sby016.519

**Published:** 2018-04-01

**Authors:** Nils Sjöström, Mats Ewertzon, Bente Weimand, Anita Johansson, Zophia Mellgren, Ola Johansson, Jane Ek-Persson, Margda Waern

**Affiliations:** 1 Sahlgrenska University Hospital; 2 Ersta Sköndal Bräcke University College; 3 Akershus University Hospital; 4 Skaraborgs Hospital;; 5 Dish Association of Local Authorities and Regions; 6 Sahlgrenska University Hospital, University of Gothenburg

## Abstract

**Background:**

Relatives often take on great responsibility for helping the patient in his or her daily life, and many relatives experience lack of support from health care services. Cooperation with relatives is a central component in Resource groups Assertive Community Treatment (R-ACT). This person-centered model has been found to decrease symptoms, increase level of function, and strengthen well-being in patients with psychotic disorders. However, little is known about relatives’ experiences of the model.

**Aim:**

To examine relatives experiences of R-ACT. Further, to compare relatives’ experiences of treatment and feelings of being alienated from care services in relatives’ with and without experience of R-ACT. We hypothesize higher levels of family burden, and family stigma and lower quality of life in relatives without R-ACT.

**Design:**

Cross-sectional study focusing on relatives of persons with psychotic disorders during the period of October 1, 2017 – May 31, 2018.

**Participants:**

Relatives of next of kin suffering from psychotic disorders, treated in health care clinics with and without R-ACT in Västra Götaland County in Sweden.

**Measurements:**

The postal questionnaire includes four self-reported instruments: the Family Involvement and Alienation Questionnaire, the Burden Inventory for Relatives of Persons Psychotic Disturbances, the Inventory of Stigmatizing Experiences (family version), and RAND-36.

**Results:**

Recruitment is ongoing. Preliminary results will be presented at the conference.

**Discussion:**

Increased knowledge about relatives’ experiences of psychosis care can inform the development of R-ACT, a care model that focuses on participation of both patients and their relatives.

